# Supplementary material for: Effectiveness of community-based health education and home support program to reduce blood pressure among patients with uncontrolled hypertension in Nepal: A cluster-randomized trial
Source: PLoS One. 2021 Oct 12;16(10):e0258406. doi: 10.1371/journal.pone.0258406 (PMC8509872; doi:10.1371/journal.pone.0258406)
Supplement: S1 Table — (DOCX) [file pone.0258406.s001.docx]

| **Contents of health education sessions** | | | | | |
| --- | --- | --- | --- | --- | --- |
|  | Chapter | Contents | Method | Media/tools | Time |
| 1 | Communication with the participants | Establishing Communication and identifying barriers and rapport, elicit personal views on hypertension and its treatment, contextual influences on hypertension management (social, cultural, migration and finance) | Interactive communication |  | 15 min |
| 2 | Introduction of blood pressure | Understanding, measurement and meaning of numbers of blood pressure, healthy and unhealthy blood pressure range, burden of hypertension in Nepal | Interactive lecture | Drawing paper  Pictures  Marker | 15 min |
| 3 | Causes of high Blood pressure | Causes, non-modifiable risk factors, modifiable risk factors | Interactive lecture | Drawing paper  Pictures, cartoon,  Marker | 15min |
| 4 | Complication of High blood pressure | Complications of high blood pressure | Interactive lecture | Drawing paper  Picture showing complication,  Marker | 15 min |
| 5 | Lifestyle management of high blood pressure | Healthy eating, nutritional recommendation based on Dietary approaches to stop hypertension (DASH), physical activity, maintaining healthy weight , limit (or avoid) alcohol, quit tobacco, reducing stress, tips for changing daily life in context of living condition of community | Interactive lecture, experience sharing by member of hypertension controlled group | Drawing paper  Pictures, cartoon,  Marker | 30 |
| 6 | Medications of high blood pressure | Indications of antihypertensive medication, Types of medicines, mechanisms to reduce blood pressure, monitoring of blood pressure medication, ways to reduce side effects of medicine, importance of adherence of medicine | Interactive lecture, experience sharing by member of hypertension controlled group | Drawing paper  Pictures, cartoon,  Marker | 30 |
| 7 | WHO/ISH risk prediction chart for CVD | Introduction about the chart, way to use chart and calculating ten years risk, WHO recommendation for controlling blood pressure according to risk category | Interactive lecture, demonstration | WHO/ISH chart, Drawing paper | 30 |
