# Supplementary material for: Effectiveness of community-based health education and home support program to reduce blood pressure among patients with uncontrolled hypertension in Nepal: A cluster-randomized trial
Source: PLoS One. 2021 Oct 12;16(10):e0258406. doi: 10.1371/journal.pone.0258406 (PMC8509872; doi:10.1371/journal.pone.0258406)
Supplement: S2 Table — (DOCX) [file pone.0258406.s002.docx]

| S2 Table: Linear regression model for change in knowledge score | | | | |
| --- | --- | --- | --- | --- |
| Characteristics | Constant | B | 95% Confidence interval of B | P value |
| Intervention | 4.019 | 2.65 | 2.084-3.215 | **0.001** |
|  |  |  |  |  |
