# Supplementary material for: Effectiveness of community-based health education and home support program to reduce blood pressure among patients with uncontrolled hypertension in Nepal: A cluster-randomized trial
Source: PLoS One. 2021 Oct 12;16(10):e0258406. doi: 10.1371/journal.pone.0258406 (PMC8509872; doi:10.1371/journal.pone.0258406)
Supplement: S4 Table — (DOC) [file pone.0258406.s004.doc]

Research Title:

| **Effectiveness of community based health education in reduction of blood pressure among uncontrolled hypertensive patients of suburban population of Surkhet District of Nepal** |
| --- |

1. Proposal Summary (maximum 500 words):

| **Introduction** One third of death globally and 40% of admission in hospital among all non communicable diseases in Nepal is attributed to cardiovascular disease. Hypertension is important risk factor of cardiovascular disease and kidney disease. If blood pressure remains uncontrolled, chances of complication will be high among hypertensive patients. Although educating the patients about lifestyle modification and medication can contribute to reduce blood pressure, no studies has been conducted in Nepal in a community setting.  **Objective**: To evaluate the effect of health education on blood pressure control compared to usual care among uncontrolled hypertensive patients of suburban community of Surkhet district of Nepal  **Method:** Cluster randomized trial will be conducted in Birendranagar Municipality of Surkhet District of Nepal. Among 12 wards four wards will be selected randomly. Uncontrolled hypertensive patients will be screened visiting every household of the selected ward in systematic manner. Total sample size will be 152 in intervention arm (76) and control (76) arm. Total 38 eligible participants will be enrolled from each cluster who meet the inclusion criteria and provide consent. Two clusters will be assigned to provide hypertension-related health education by using a simple randomization procedure. Uncontrolled hypertensive patients from rest of two clusters will be the control group who will get usual care. Four sessions of health education will be provided to intervention group within six months. Female community health volunteers will meet the participants twice a month, record their blood pressure and counsel the respondent and his family. Follow up data collection will be conducted after six months. Paired t- test (continues variable) and Chi-square test (binary variable) will be used to test the significance of baseline and after six months for both intervention and control groups. Similarly, independent t test (continuous variable) and Chi-square test (binary variable) will be used to test the difference between the groups. Multiple logistic regression analysis will be conducted to evaluate the effect of intervention with and without adjusting confounders.  **Expected Results:** Primary outcome of the study will be the proportion of normalized systolic blood pressure in both the groups. The study will determine the effect of intervention on the between-group difference in the proportion of patients with normalized systolic blood pressure. Change in knowledge score in both groups will also be calculated after six months.  **Conclusion:** The study will be useful to evaluate the effectiveness of health education in a community setting. Thus the study will be useful for planning and implementing health education in the community to control blood pressure. |
| --- |

1. Introduction:
   1. Background of Study (maximum 500 words):

| Hypertension is an important risk factor for cardiovascular disease and kidney disease. Globally around one-third of death is attributed to cardiovascular disease. Mortality due to cardiovascular disease was 17.5 million in 2008. At least three-quarters of the world's deaths from CVDs occur in low- and middle-income countries. Among 16 million deaths under the age of 70 are due to noncommunicable diseases, 37% was due to cardiovascular disease. Hypertension is responsible for about fifty percent of mortality among the death due to cardiovascular disease worldwide . Hypertension is responsible for at least 45% of death due to heart disease and 51 % of death due to stroke.  Hypertension is a significant public health problem. It is the number one leading risk factor for global disease burden. In comparing the different regions, the leading risk factor in most of Asia, most of Latin America, North Africa and the Middle East, and central Europe it was high blood pressure . Notable shifting of high blood pressure was identified by GDB study in 2010. Hypertension was the 4^th^ leading risk factor in 1990 whereas it was the 1^st^ leading risk factor in 2010. Worldwide, 40% of adults aged 25 and above had been diagnosed with hypertension in 2008.  The prevalence of hypertension is high and had been increasing in Nepal. In STEPs surveillance 2007, 18.1 % were hypertensive that was increased to 25.2% in 2013. Study shows there has been three-fold increase in prevalence of hypertension over 25 years.This evidence shows that hypertension is a rapidly growing public health issue.  Adverse consequences of hypertension will be increased if blood pressure is not controlled in time. Risk factors like tobacco use, unhealthy diet, harmful use of alcohol and physical inactivity can compound the raised blood pressure to develop complications . Clinical randomized trials proved that lifestyle modifications and antihypertensive medication can control the blood pressure thus lower the risk of CVD. Different targeted interventions have been identified to improve adherence to these lifestyle modifications and antihypertensive medication.  Patient education will augment disease perception, motivation, and ability to adhere hypertension control goal. Different studies had been conducted in developed countries for evaluating effectiveness of health education of the hypertensive patients. However, in developing countries like Nepal, there is lack of evidence that the health education can establish the disparities in controlling blood pressure. |
| --- |

- 1. Statement of the Problem and Rationale / Justification (maximum 500 words)

| Uncontrolled hypertension is the major public health problem among hypertensive patients in Nepal. One-fourth adult population aged 15 to 65 years is hypertensive. According to a national study of STEPS surveillance, the proportion of hypertensive patients with normalized blood pressure are only 3.8%.However, most of the hypertensive patients (88.3%) had not started the antihypertensive medication. Eight percent of hypertensive patient did not have controlled blood pressure even after taking medicine. Among previously diagnosed hypertensive patients, almost half patients are not taking antihypertensive medication even though doctor or health worker prescribed them. But, most of the previously diagnosed patients were not getting advice to reduce salt intake (25%), to start or more exercise (65%), to lose weight (60%) and to stop smoking (38%) (STEPS 2013).  Adherence to antihypertensive medication and lifestyle modifications including decreased sodium intake, weight loss, increased physical activity, reduced excessive alcohol intake, increased potassium intake, consumption of healthy diet like fruit and vegetable, low-fat dairy products and reduced saturated and total fat and lifestyle modification is mandatory to control the blood pressure. Patients' non-adherence to therapy is increased by lack of knowledge on benefit for preventing complications, lack of motivation, low perceived risk of high blood pressure, denial of illness because of lack of symptoms, disbelief in treatment, fear of unexpected adverse effects of medications or drugs, out of pocket medication cost, lack of immediate effect by lifestyle interventions. Patient education will improve knowledge on hypertension, its complications, and benefit of early treatment and control along with adherence to medication and change in lifestyle. In one study, after six months of hypertension education, mean SBP/DBP had dropped by 10/5.7 (SD14.3/9.2) mmHg in intervention group and by 6.3/1.7 (SD 13.4/8.6)mmHg in the control group. Mean scores for adherence to lifestyle recommendations increased in the intervention group, but decreased in the control group .  Nepal is suffering from double burden of diseases. In a hospital based study one third were admitted for noncommunicable diseases, among them 40% had cardiovascular problem. Hypertension is the important risk factor of cardiovascular diseases. Behavior change is important to control the risk factors of noncommunicable diseases. Educating the patient and population about the importance of healthy behavior is the first step to behavior change. Despite Nepal has recently promulgated new health policy focusing on health education for prevention and control, no study has been conducted yet to evaluate effectiveness of community-based health education along with support from their community to control the blood pressure.  Thus, we seek to screen uncontrolled hypertensive patients in community, then provide interactive community based health education along with support from the community volunteers so that the people will be enabled to control their own blood pressure. Therefore, the study will guide to design and implement policy for health education in community of Nepal. |
| --- |

- 1. Conceptual framework

**Health Education**

Normalized systolic blood pressure (SBP<140 mmHg)

Increase physical activity, fruit and vegetable intake, reduce weight, stop smoking and alcohol

Adherence to medication

Increase knowledge related to hypertension

Perceive the risk of hypertension, understand the role of modifiable risk factors and benefit of healthy behaviors

Confounders:

Age

Sex

Level of education

Occupation

Income

Duration of hypertension

Number of Medication

Diabetes mellitus

- 1. Research Objectives / purpose / aim of the study:

General

| To evaluate the effect of health education compared to usual care on blood pressure control among uncontrolled hypertensive patients of suburban community of Surkhet district of Nepal |
| --- |

Specific

| To determine the proportion of patients with normalized systolic blood pressure after six months of health education intervention and usual care among uncontrolled hypertensive patients of suburban community of Surkhet district of Nepal  To determine the effect of intervention on the between-group difference in the proportion of patients with normalized systolic blood pressure among uncontrolled hypertensive patients of suburban community of Surkhet district of Nepal  To determine the change in knowledge score related to hypertension after six months of health education intervention and usual care among study population.  To determine the change in adherence of lifestyle modification after six months of health education intervention and usual care among study population. |
| --- |

1. Research Design and Methodology

Research Method


Qualitative Quantitative Combined

## **Study variables**

**1)Demographic variables:** Age, Sex, Ethnicity/cast, Marital Status, Level of Education, Employment/Occupation and Monthly Income per person per month. **2)Behavior Related variables:** Current smoker, number of sticks per day, Past smoker, Current drinkers, Amount of standard drinks per day, servings of fruit and vegetables per day, average amount of salt intake per day, Level of physical activity **3) Variables related to hypertension:** Duration of hypertension, adherence score of lifestyle modification, history of antihypertensive drug intake, number of antihypertensive drugs used, knowledge score related to hypertension, medication adherence **4) Physical Measurements:** Height, Weight, BMI, Waist circumference, Hip Circumference, SBP,DBP **5)Other variables:** Family history of hypertension

## **Type of study:**

Cluster randomized controlled trial

## **Study Site and Justification:**

Study will be conducted in Birendranagar Municipality of Surkhet district of Nepal. The Surkhet district is located about 600 Km west of the national capital Kathmandu. Birendranagar is a valley and the largest suburban city of that region, which is hub to remote districts like Dailekh, Kaalikot, [Jumla](https://en.wikipedia.org/wiki/Jumla_District),Humla,  Dolpa, [Mugu](https://en.wikipedia.org/wiki/Mugu_District), [Jajarkot](https://en.wikipedia.org/wiki/Jajarkot), and [Salyan](https://en.wikipedia.org/wiki/Salyan_District,_Nepal). Recently because of insurgency, many people have migrated to Surkhet from these districts. Total population of the Municipality is 47914. It has twelve wards. Half of the inhabitants of the city are high cast groups like Bramhin, Kshetri, rest are mostly indigenous and low cast groups like Damai, Kami, Sunar .The city has one regional hospital for tertiary care, three private nursing homes, one zonal Ayurveda Hospital and about 20 dispensaries. Unpublished and observed data of these institutions shows increased numbers of uncontrolled hypertension. Therefore, we want to conduct an educational intervention program focused in the community so that people will be motivated to adhere lifestyle modifications and medication together.

**Study population**: Uncontrolled hypertensive patients of age ≥ 30 years, who have SBP≥140 mmHg and or DBP≥ 90 mmHg, are living for at least one year and will live at least for another six months in Birendranagar Municipality of Surkhet district of Nepal.

## **Study unit:** Uncontrolled hypertensive patients of age ≥ 30 years will be study unit.

**Sampling method/techniques:**

Two stage sampling technique will be used.

- **First stage:** Eligible participants will be identified using clustered sampling technique. Ward will be a cluster. Among twelve wards of the municipality, thirty percent of the ward (four wards) will be chosen randomly.
- **Second Stage:** After the completion of participant recruitment and baseline data collection in all four clusters, clusters will be randomly assigned to intervention groups. Among four wards (n=4) two wards will be selected randomly (using computer generated sequence number) for health education intervention group (n=2) and two wards for usual care control group (n=2). Usual care will get standard hypertension care as prescribed by their health professionals. Randomization of cluster instead of individual can minimize the contamination of health education intervention between the clusters.

## **Sample size calculation:**

Primary outcome is proportion of patients with normalized systolic blood pressure of less than 140 mm Hg. Systolic is the upper level and easy to remember for the patient. Total sample size will be 152. It is estimated that normalized blood pressure will be achieved in 25% of uncontrolled hypertensive patients after health education while only 5.2 % uncontrolled hypertensive patients achieve controlled blood pressure through usual care achieving 19.8% difference after intervention . Considering that the different clusters are in same geographical area having similar culture we assume intra-cluster correlation (ICC) coefficient of 0.03 based on literature review. We assume two-sided t-test of 5% and 80% power. Using the formula for individual randomization:

ni= (z_ἁ/2_+z _ᵦ/2_)2 (p1q1+p2q2)/ (p1-p2)^2^ =48

Assuming the equal sized clusters in the community required number of clusters in each arm is two. Increase in sample size over that required under individual randomization to achieve the power equivalent to individual randomization is 18 using a formula after considering fixed number of cluster .

ni*(1-icc)/(k-ni*icc)=18

We expect dropout rate of 15% in each arm, thus total sample size per arm will be 76. We will screen in all four clusters to get at least 50 uncontrolled hypertensive patients (40% more) from each cluster before final assignment into intervention and usual care group.

## **Inclusion criteria:**

- Who is willing to participate and give consent for the study
- Uncontrolled hypertension (SBP≥140 mmHg and/or DBP≥90mmHg) including those on antihypertensive medication
- Age ≥30 years
- Can understand Nepali language
- Capable of effective oral communication
- Who has telephone or mobile number

## **Exclusion criteria:**

- Dementia and other mental illness
- Bed ridden patients
- Known kidney disease, cancer, heart problem, chronic obstructive pulmonary disease
- Pregnant women
- Participant who is either blind or deaf

## **Data collection techniques:**

- **Interview:** Face to face, Interview will be taken by trained interviewer using Standard STEPS instrument version 2.2 along with separate questionnaires for knowledge related to hypertension and adherence to medication.
- **Physical measurements:** All physical measurements will be taken in a separate room. Blood pressure, height, weight, waist circumference and hip circumference will be measured in sequence.
- **Blood pressure measurement:** For measuring blood pressure doctors' sphygmomanometer and stethoscope will be used with appropriate sized cuff. The first reading will be taken after requesting participants to take 15 min rest crossing leg. The second reading will be taken at three minutes rest after first reading. Sphygmomanometer will be placed on the unclothed left arm above the elbow while putting the arm in the table facing palm up. Mean of two readings will be used for final assessment.
- **Height measurement:** Height will be measured in a portable standard height measuring scale. Height will be measured without head gear or foot bear. The participants will be requested to stand on the flat surface, with straight knee, feet together and heels against the backboard. It will make sure that respondent is looking straight to the interviewer, not tilt their head up and eyes and ears at same level.
- **Weight:** Weight will be measured using a portable weighing scale. The scale will be put in a firm flat surface. The participant will be requested to step onto the scale only wearing light cloths removing foot wear and socks. Then standing straight, facing forward and placing arms on the side.
- **Waist and hip circumference measurement:** Waist and hip circumference using a constant tension tape. A private area, such as a separate room within the house, will be used and the measurement will be taken over light clothing. Waist circumference will be taken at the end of a normal expiration with the arms relaxed at the sides at the midpoint between the lower margin of the last palpable rib and the top of the iliac crest (hip bone). Hip circumference will be taken at the maximum circumference over the buttocks. Participants will be requested to wrap the tape around themselves. Measurement will be taken at the nearest 0.1 cm, making sure to keep the measuring tape snug.

## **Data collection tools:**

- STEPs questionnaire: This questionnaires contains questions related to socio-demographic, smoking, alcohol, fruit and vegetable intake, level of physical activities, history of high blood pressure, diabetes, heart diseases, kidney diseases and physical measurements.
- Tool to assess knowledge related to hypertension: This tool has ten questionnaires, where six questionnaires have three multiple choice answers and four questionnaires have more than three multiple choice answers. Only one answer should be chosen by the respondent. Score one will be given for a correct answer and a score zero will be given for incorrect, don’t know and not sure answers where as a score zero point five will be given to partially correct answers .
- Tool for medication adherence: Adherence to medication will be assessed using eight-item Morisky Medication Adherence Score (MMAS-8). The scale has seven questions with 'yes'/'No' answers and one five-point Likert scale question. The total score will be eight where higher score will be good adherence lower scale represents poor adherence. The tool is translated in Nepali by two separate investigators and then compiled into final version by principle investigator.
- Stethoscope
- Sphygmomanometer
- Weighing machine
- Height stand

## **Data Collection Procedure:**

Data collection will be finished in following steps:

**Step one**: Trained enumerator, blinded for randomization status, will visit household of the selected wards. At first, one corner of the ward will be visited randomly. Then nearby road and then right or left side of the road will be chosen randomly. The first household of that road will be taken as first household to be visited. Then, then every household will be visited to screen for uncontrolled hypertensive patients until we get 50 such patients in each cluster. Every member of the household above the age of 30years who is not bed-ridden, not having mental illness, not pregnant and without known heart problem, kidney disease, chronic obstructive pulmonary diseases, and who provide consent will be screened using STEPS questionnaire. Before taking consent, objectives, methods, benefit and risk of the study will be explained. Exclusion of the diseased person will be based on the respondents' reply. During this screening visit, enumerator will record detail of household information, mailing address, and telephone/mobile number of the participant, along with demographic information, smoking and alcohol drinking status, fruit and vegetable intake and level of physical activity along with anthropometric measurements. Then blood pressure and BMI status of the respondent will be informed to the patient. Finally, knowledge related to hypertension and medication adherence will then be taken using questionnaires from the uncontrolled hypertensive patient. The enumerator will immediately refer to a nearby hospital if any respondent has malignant hypertension of SBP/DBP ≥ 180/110 mm Hg.

**Step two:** A separate team will check the data of the respondents. The team will select the eligible patient who meets the inclusive criteria. Then, we will send a letter to remind the eligible patients who meet the inclusion criteria with information about the study and to thank for participation in the study. The enrollment of the participant will be re-confirmed through telephone. Then, all the baseline information of the participants who will be enrolled in the study will be saved. By these procedures, required number of patients in all four clusters will be recruited and get the baseline data.

**Step three**: Randomization of the cluster will be done after completion of the baseline data. A separate team not involved in baseline data collection will use the computer-generated randomization to allocate the clusters into health education intervention and usual care. For intervention, four groups containing 15-20 participants will be formed, based on the geographic location of the participants. Finally interactive lecture date for health education, venue and time will be informed to the respective groups by telephone. Four sessions of health education will be given to each of the intervention groups.

**Step four:** Follow up information will be taken after six months from patients of intervention and control clusters by same tool used in baseline. Separate enumerator who are blinded to intervention and control group will take the final data.

**Step five:** Similar intervention will be provided to the control group if we get the expected benefit from previous six months study.

## **Intervention:**

- Usual care will be provided to the control group. Usual care is the standardized care provided by their health professionals available within the municipality. Interactive health education lectures along with support from the community volunteer will be provided to the interventional group.
- **Interactive Health education lectures:** Total four interactive lecture sessions with two hours of duration will be conducted for each group. First lecture at the start of the study, second at one month of the study, a third class at three months of the class and last one will be at fifth month of the study. The session will be based on the syllabus (annex V). Syllabus has been designed based on patient information by American Heart Association and the seventh report of the joint National Committee on Prevention, Detection, evaluation, and treatment of high blood pressure. Tools prepared by the National Health Education, Information and Communication Centre of Department of Health Services of Nepal will also be used as education materials. Education material for WHO/ ISH risk prediction chart will be based on Prevention of cardiovascular disease: guidelines for assessment and management of total cardiovascular risk (WHO, 2007). Health education syllabus is comprised of seven chapters including communication, introduction to hypertension, causes, and complication of high blood pressure, lifestyle management, medication for hypertension and WHO/ISH risk prediction chart for development of cardiovascular disease within 10 years. One trained nurse and one medical graduate will facilitate lecture session for each group. In first session, we will focus on first four chapters and go briefly on fifth and sixth chapters. Second session will start after brief communication of previous knowledge, experiences, and barriers based on previous session then go in detail about the chapter of fifth, sixth and seventh chapters. In third and fourth session, we will make two separate set of 8-10 participants from each group, check log book prepared by community volunteers and make interactive discussion regarding their experience on changing diet, increasing physical activities, maintaining body weight, adherence to medications, side effects, problem, and difficulties on changing behavior, any taboos and misconception, encouraging and facilitating to the patients through the health educator and hypertension control volunteer.
- **Community support through FCHV:** At least one female community volunteer for each cluster will be mobilized for motivating and supporting the participants in the community. Separate training for accurate blood pressure measurement and behavior change counseling will be provided to community volunteers based on same syllabus designed for participants. One set of sphygmomanometer and stethoscope and log book will be provided to these volunteer. These volunteer will also attain the health education session along with the participants. She will visit the household of the participant twice a month, measure and record the blood pressure in a log book then discuss the problems facing personally or in family, culture in controlling blood pressure, and take feedback from the participants. If suspects any serious health-related problem, malignant hypertension of SBP/DBP ≥180/110 mm Hg, she will advise to visit neighborhood health care facilities. Discuss with the family members about the behavior that should be changed to control blood pressure. Educate to the family member responsible for cooking about benefits and ways of salt reduction and DASH diet and to remind for drug intake daily. This will help to remind for adherence and then boost the confidence among the participants.

## Pre-testing the Data Collection Tools:

Pre-testing of data collection tools will be conducted in 15 samples in cluster not selected for the study. Then questionnaires and tool will be modified before final implementation of the research.

## Validity and Reliability of the Study Tools:

Although pre-testing of STEPs 2.2 version tool is already validated, tool for evaluating knowledge related to hypertension and medication adherence is not validated in Nepal in current setting.

## Potential Biases:

As the study will need to select for intervention and control group, selection bias and measurement bias will be the most threatening issues. Separate trained field workers will be involved in screening and baseline data collection and final follow up data collection in all four clusters. After completion of baseline data collection a team not involved in data collection and management will randomize clusters for the intervention and control group. These steps will be useful to minimize the selection and measurement bias.

## Limitations of study:

- Study is limited to single and selected community having population of different geographic area, cast and culture. Thus, the study should cautiously be generalized to other communities.
- As no similar type was conducted in Nepal and India, sample size calculation is being difficult. We have estimated small sample size based on a study from China with different settings and different duration of study, which may not be enough sample size to evaluate the effectiveness of health education. But, it is not possible to increase the sample size based on the resource available.
- We are going to randomize the cluster instead of individual randomization. Even though we are adjusting the design effect to conclude the result at individual level, power may not be equivalent to a study with individual randomization.
- As intervention will be provided at cluster level in group, it will be difficult to conceal the intervention status to the enumerator during follow up data collection. But, employing the trained enumerator not involved in baseline data collection will distribute the bias equally to both groups.
- Tool for measuring knowledge related to hypertension and medication adherence yet to be validated in Nepal.

1. Plan for Supervision and Monitoring:

Supervision and Monitoring will be conducted principle investigator and co- investigators.

1. Plan for Data Management and Analysis:

Data will be compiled, edited and checked to maintain consistency for all data of screening, baseline and follow up stage. Repetitions and omissions of data will be corrected before coding and entering them in Epi-Info. Recorded data will be, then, exported to SPSS V.16.0 for further analysis. Data will be analyzed by a statistician blinded to the intervention status. Analysis of the trial will be done for the individual participants who have attended at least three sessions of health education and will be followed up.

Descriptive statistics will be used to present the characteristics of the study subjects for both baseline and follow up stage. Proportion of normalized blood pressure, mean of knowledge score related to hypertension, proportion of smoking and alcohol, mean servings of fruit and vegetable, mean of salt intake per day, level of physical activity in METS, mean of adherence score of medication will be calculated for both the intervention and control group separately. Paired t- test (continues variable) and Chi-square test (binary variable) will be used to test the significance for baseline and after six months for each group. Similarly, independent t test (continuous variable) and Chi-square test (binary variable) will be used to test the difference between the groups. Multiple logistic regression will be used with dependent variable as presence or absence of normalized blood pressure then effect of intervention will be calculated as odds ratio after adjusting age, socioeconomic status, and clustering effect. All tests will be two-tailed and p < 0.05 will be considered statistically significant. Data will be presented in frequency tables, diagrams and bar.

1. Expected Outcome of the Research:

Primary outcome of the research will be proportion of hypertensive patients with normalized blood pressure in intervention and control group. While secondary outcome will be to change in knowledge score, change in adherence of lifestyle modification after six of months of health education and usual care. This result will provide the evidence of education to the hypertensive patients along with support from the community volunteer to manage the blood pressure. Thus, the study helps to support for the national policy development.

1. Plan for Dissemination of Research Results:

Research Dissemination will be in two steps. First report will be disseminated after getting the screening report for hypertensive patients. Second report will be disseminated after completion of the intervention. Dissemination will be through community gathering, local media and through journal article.

1. Plan for Utilization of the Research Findings (optional):

Research finding can be utilized for development of national strategy and policy development to control hypertension and risk of cardiovascular diseases.

How is the research project going to strengthen the research capability of the host institution: Nepali Researcher (if submitted from abroad):

NA

**Part – IV**

**Ethical Consideration**

22. Regarding the human participants:

Are human participants required in this research? If yes, provide justification.

Yes (*provide justification*) No

We are going to conduct hypertension related health education

How many participants are required for the research? Explain.

| Total 152 uncontrolled hypertensive patients will be required for intervention and control group |
| --- |

What is the frequency of the participant’s involvement in the research? Explain.

| Participants' data collection will be conducted two times. One at the baseline and then after six months. |
| --- |

Clearly indicate the participant's responsibilities in the research. What is expected of the research participants during the research?

| Participants will give the answer to questions; give the anthropometric measurement and blood pressure. |
| --- |

Are vulnerable members of the population required for this research? If yes, provide justification.

| NA |
| --- |

Are there any risks involved for the participants? If yes, identify clearly what are the expected risks for the human participants in the research and provide a justification for these risks.

| NA |
| --- |

Are there any benefits involved for the participants? If yes, identify clearly what are the expected benefits for the participants.

| Yes, participant will get knowledge related to hypertension, know ways to control blood pressure. |
| --- |

23. Informed Consent Form / Ethical Issues:

*(Informed Consent form should be submitted in English and in the language appropriate to the research participants)*

Obtaining the Consent

How informed consent is obtained from the research participants?

Verbal Written

Please indicate who is responsible for obtaining informed consent from the participants in this research study?

- *Enumerator will be responsible for obtaining informed consent*

Is there anything being withheld from the research participants at the time the informed consent is being sought? NA

If yes, explain ……………………………………………………………………………

Is the research sensitive to the Nepali culture and the social values?

Yes No Explain.

- *We seek to change the lifestyle, feeding habit and misconceptions regarding hypertension*.

Is health insurance *(if applicable)* being made available to the research participants? If yes, please provide the necessary insurance data.

- *NA*

(Include in consent form)

24. Regarding Clinical Trial:

In case of a clinical trial address the following:

- *The trial is going to provide hypertension related health education.* ……………………………………………………………………………

……………………………………………………………………………

The potential or direct benefits (if any) for the research participants.

- *Participant will get knowledge related to hypertension, know ways to control blood pressure.* ……………………………………………………………………………

Alternative procedure(s) or treatment(s) that may be available.

……………………………………………………………………………

The risks, discomforts, and inconveniences associated with the study

- *There will be no risk of the study. Discomfort will be attending the health education session and need to manage time from the participants.*

Provisions for management of any adverse reactions

- NA

The provisions of insurance coverage for any permanent disability or death caused directly by the investigational treatment or procedure.

- NA

The provision of including the name and address, including telephone numbers of person to be contacted in case of adverse events or for any information related to the trial.

- NA

Is there going to be a transfer of any biological materials from the country? Explain.

- NA

Is there a Data Safety Monitoring Board?

If Yes, Mention

- NA

Is this trail internationally registered?

- Not registered

**References**
