# Supplementary material for: Effectiveness of community-based health education and home support program to reduce blood pressure among patients with uncontrolled hypertension in Nepal: A cluster-randomized trial
Source: PLoS One. 2021 Oct 12;16(10):e0258406. doi: 10.1371/journal.pone.0258406 (PMC8509872; doi:10.1371/journal.pone.0258406)
Supplement: S5 Table — (DOCX) [file pone.0258406.s005.docx]

| S5 Table: Distribution of mean difference at 6-month follow-up SBP and DBP by gender, age, in both control and intervention group | | | | | | | |
| --- | --- | --- | --- | --- | --- | --- | --- |
|  |  |  | **Control (n=60)** | | **Intervention (n=60)** | | **P value** |
| Variables | |  | **Number (n)** | **Mean Difference (SD)** | **Number (n)** | **Mean Difference (SD)** |  |
| SBP |  |  |  |  |  |  |  |
|  | **Gender** | **Male** | 27 | 12.2 (20.8) | 27 | 12.2 (14.5) | 0.991 |
|  |  | **Female** | 33 | 10.3 (19.8) | 33 | 24.1 (22.1) | **0.010** |
|  | **Age** | **30-44** | 8 | 18.7 (17.0) | 9 | 10.2 (7.7) | 0.196 |
|  |  | **45-59** | 26 | 10.3 (18.3) | 23 | 23.4 (23.2) | **0.033** |
|  |  | **>60** | 26 | 9.8 (22.7) | 28 | 17.7 (18.9) | 0.170 |
|  | **Total** |  | 60 | 11.2 (2.5) | 60 | 18.7 (19.8) | **0.041** |
| DBP |  |  |  |  |  |  |  |
|  | **Gender** | **Male** | 27 | 5.1 (16.2) | 27 | 10.5 (13.0) | 0.186 |
|  |  | **Female** | 33 | 5.8 (18.0) | 33 | 11.3 (16.0) | 0.200 |
|  | **Age** | **30-44** | 8 | 7.5 (18.3) | 9 | 7.3 (9.7) | 0.981 |
|  |  | **45-59** | 26 | 7.7 (18.8) | 23 | 12.6 (17.1) | 0.346 |
|  |  | **>60** | 26 | 2.6 (15.1) | 28 | 10.6 (13.9) | 0.049 |
|  | **Total** |  | 60 | 5.53 (2.2) | 60 | 10.95 (14.6) | 0.065 |
