# Supplementary material for: Effectiveness of community-based health education and home support program to reduce blood pressure among patients with uncontrolled hypertension in Nepal: A cluster-randomized trial
Source: PLoS One. 2021 Oct 12;16(10):e0258406. doi: 10.1371/journal.pone.0258406 (PMC8509872; doi:10.1371/journal.pone.0258406)
Supplement: Attachment — Submitted filename: Response to Reviewer 1.docx [file pone.0258406.s006.docx]

Reviewer 1

General comments:

Authors reported that multiple health education sessions complemented by frequent household visits by health volunteers can effectively improve knowledge on hypertension and reduce the blood pressure among uncontrolled hypertensive patients at community level in Nepal. This manuscript may be interesting. However, there are several concerns in this manuscript.

To make all data fully available, we have provided a temporary link: <https://datadryad.org/stash/share/1TgkHL_KKAiAums8pRV6rj52MC_3DO7lukcXvDA1H0k>.

Specific Comments:

| SN | Major Comments | Response |
| --- | --- | --- |
| 1 | The most serious concern may be a small number of subjects as authors raised it in the section of limitation. The small sample size will lead a wrong conclusion. | Authors have already acknowledged that we have a limitation of small sample size and suggested to carefully interpret the results. We wrote this "we could use only a small sample size. This implies the result must be interpreted carefully" on the manuscript. Our study is first in the region where we had conducted the trial. Our findings could provide a few evidence to carry out a large, multicentre randomized trial with similar intervention, which can provide the definitive conclusion.  Data from small studies can be used to design larger confirmatory studies [1, 2]. |
| 2 | In Tables 3 and 6, authors should not analyze data by adjusting for SBP and DBP simultaneously. | In both the tables, we have separately adjusted for SBP and DBP for controlled systolic blood pressure and controlled diastolic blood pressure, respectively. However, we adjusted for SBP and DBP for controlled BP (both SBP and DBP simultaneously.    We have clarified this in the data analysis subheading of method section. |
| 3 | In Tables 3-5, how do authors deal with the use of antihypertensive medication, which may impact on systolic and diastolic blood pressures? | In our study, no participants reported any changes in medication use. We had already mentioned this in the line 287 of the result section. Therefore, we have confidence that the antihypertensive medication did not affect our study findings. However, we could have assessed the drug adherence, we have already mentioned this in limitation section. |
| 4 | The section of “Results” is too long and redundant. | To address this, we have shifted table 5 in supplementary file. Now, we have only five tables in the result section. A few redundant sentences are removed. |
| 5 | Authors should ask natives to edit English. | We have corrected accordingly. |
|  | Minor Comments |  |
| 1 | Although systolic blood pressure and diastolic blood pressure were already abbreviated as SBP and DBP, authors were using systolic blood pressure and diastolic blood pressure without abbreviation again and again. | It is corrected in the manuscript. |
| 2 | In the footnote of Table 1, Fischar exact test may be wrong. Fisher’s exact test may be correct. Authors should add the explanation in the section of data analysis. | Corrected |
| 3 | In the section of result, Effectiveness of intervention: line 312; “After adjusting for” is correct. Line 316; “we adjusted for” is correct. | Corrected |
| 5 | In the section of result, Sensitivity analysis: not “Odds Ratio” but “odds ratio.” Line 353; “After adjusting for” is correct. | Corrected |
| 6 | In the section of Discussion, line 380: not “8.2m Hg” but “8.2mmHg”. | Corrected |
| 7 | The journal names of the references should be accurately abbreviated | A few errors of references are corrected. |

1.	Hackshaw A: **Small studies: strengths and limitations**. *European Respiratory Journal* 2008, **32**(5):1141-1143.

2.	. In: *Small Clinical Trials: Issues and Challenges.* edn. Edited by Evans CH, Jr., Ildstad ST. Washington DC: 2001 by the National Academy of Sciences; 2001.
