# Supplementary material for: Effectiveness of community-based health education and home support program to reduce blood pressure among patients with uncontrolled hypertension in Nepal: A cluster-randomized trial
Source: PLoS One. 2021 Oct 12;16(10):e0258406. doi: 10.1371/journal.pone.0258406 (PMC8509872; doi:10.1371/journal.pone.0258406)
Supplement: Attachment — Submitted filename: Respone to Reviewer 2.docx [file pone.0258406.s007.docx]

Response to reviewer 2:

**General comments:**

A few comments were not addressed.

| **SN** | **Minor comments** | **Response** |
| --- | --- | --- |
| 1 | Page 5; line 58: not “10・8” but ”10.8” | It is corrected. |
| 2 | Page 24; line 392-398: not “6・47, 2・85” but “6.47, 2.85” | It is corrected |
| 3 | Page 24; line 393,394, 397, 398: not “mm-Hg” but “mmHg”. | It is corrected |
